# 7-Bromo-9-(2-hy­droxy-4,4-dimethyl-6-oxocyclo­hex-1-en-1-yl)-3,3-dimethyl-2,3,4,9-tetra­hydro-1*H*-xanthen-1-one

**DOI:** 10.1107/S1600536812021034

**Published:** 2012-05-12

**Authors:** Shaaban K. Mohamed, Mehmet Akkurt, Antar A. Abdelhamid, Phillip E. Fanwick, Herman Potgeiter

**Affiliations:** a Chemistry and Environmental Division, Manchester Metropolitan University, Manchester M1 5GD, England; b Department of Physics, Faculty of Sciences, Erciyes University, 38039 Kayseri, Turkey; c Department of Chemistry, Purdue University, W. Lafayette, IN 47907, USA; d School of Research, Enterprise & Innovation, Manchester Metropolitan University, Manchester M1 5GD, England

## Abstract

In the xanthene ring system of the title compound, C_23_H_25_BrO_4_, the 4*H*-pyran ring is almost planar [maximum deviation = 0.040 (3) Å] and the cyclo­hexene ring adopts a sofa conformation. The cyclo­hexene ring attached to the xanthene system is puckered [*Q*_T_ = 0.427 (3) Å, θ = 55.0 (4) ° and ϕ = 164.4 (6) °]. In the crystal, mol­ecules are linked to each other by O—H⋯O and C—H⋯O hydrogen bonds.

## Related literature

For the biological and pharmaceutical properties of xanthenes, see: Mohamed *et al.* (2012 ▶); Hilderbrand & Weissleder (2007 ▶); Shchekotikhin & Nikolaeva (2006 ▶); Fan *et al.* (2005 ▶). For related structures, see: Abdelhamid *et al.* (2011 ▶); Mohamed *et al.* (2011 ▶); Reddy *et al.* (2009 ▶); Çelik *et al.* (2009 ▶). For puckering parameters, see: Cremer & Pople (1975 ▶).
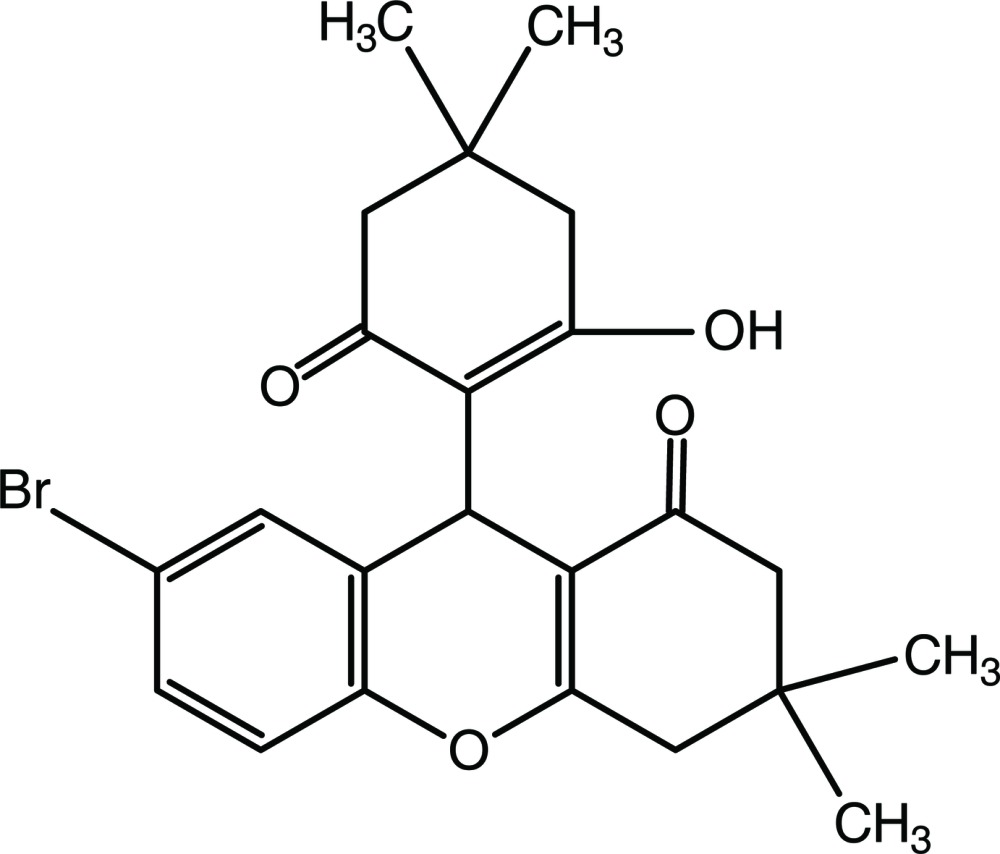


## Experimental

### 

#### Crystal data

C_23_H_25_BrO_4_
*M* *_r_* = 445.33
Orthorhombic, 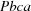

*a* = 15.6869 (4) Å
*b* = 11.0215 (2) Å
*c* = 23.0217 (16) Å
*V* = 3980.3 (3) Å^3^
*Z* = 8
Cu *K*α radiation
μ = 3.04 mm^−1^
*T* = 150 K
0.12 × 0.08 × 0.02 mm

#### Data collection

Rigaku RAPID II diffractometer
Absorption correction: multi-scan (*CrystalClear*; Rigaku, 2001) ▶ *T*_min_ = 0.712, *T*_max_ = 0.942
17325 measured reflections
3507 independent reflections
2728 reflections with *I* > 2σ(*I*)
*R*_int_ = 0.044

#### Refinement

*R*[*F* ^2^ > 2σ(*F* ^2^)] = 0.042
*wR*(*F* ^2^) = 0.107
*S* = 1.07
3507 reflections
260 parameters
1 restraint
H atoms treated by a mixture of independent and constrained refinement
Δρ_max_ = 0.46 e Å^−3^
Δρ_min_ = −0.49 e Å^−3^

### 

Data collection: *CrystalClear* (Rigaku, 2001) ▶; cell refinement: *CrystalClear*; data reduction: *CrystalClear*; program(s) used to solve structure: *SHELXS97* (Sheldrick, 2008 ▶); program(s) used to refine structure: *SHELXL97* (Sheldrick, 2008 ▶); molecular graphics: *ORTEP-3 for Windows* (Farrugia, 1997 ▶) and *PLATON* (Spek, 2009 ▶); software used to prepare material for publication: *WinGX* (Farrugia, 1999 ▶) and *PLATON*.

## Supplementary Material

Crystal structure: contains datablock(s) global, I. DOI: 10.1107/S1600536812021034/hg5218sup1.cif

Structure factors: contains datablock(s) I. DOI: 10.1107/S1600536812021034/hg5218Isup2.hkl

Supplementary material file. DOI: 10.1107/S1600536812021034/hg5218Isup3.cml

Additional supplementary materials:  crystallographic information; 3D view; checkCIF report

## Figures and Tables

**Table 1:** Hydrogen-bond geometry (Å, °)

*D*—H⋯*A*	*D*—H	H⋯*A*	*D*⋯*A*	*D*—H⋯*A*
O4—H4*O*⋯O2^i^	0.80 (3)	1.86 (3)	2.650 (3)	170 (3)
C8—H8*A*⋯O3^ii^	0.99	2.54	3.514 (4)	167

Symmetry codes: (i) 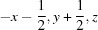; (ii) 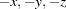.

**Fig. 1.**
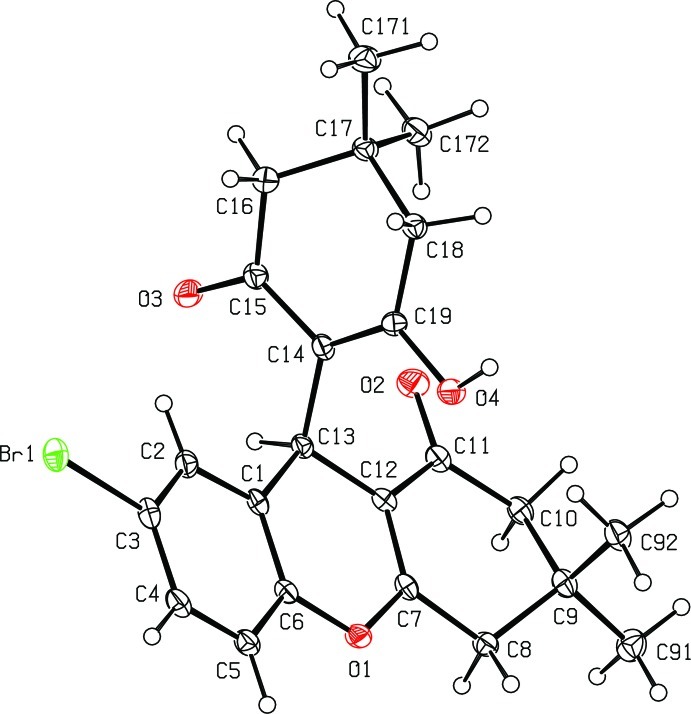
View of the title molecule with the atom numbering scheme. Displacement ellipsoids for non-H atoms are drawn at the 30% probability level.

**Fig. 2.**
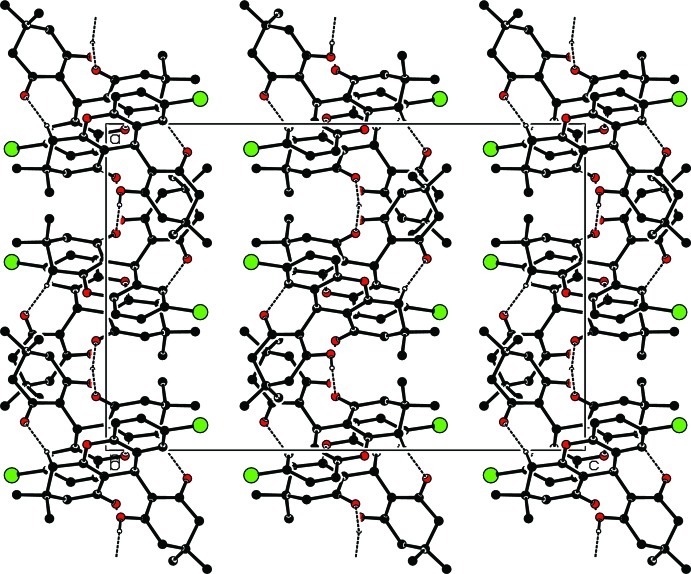
The crystal packing of the title compound, viewing along the b axis. Dashed lines show the intermolecular hydrogen bonding interactions. H atoms not involved in hydrogen bonds have been omitted for clarity.

## supplementary crystallographic information

### Comment

Xanthenes are an important class of organic compounds that received considerable attention from many pharmaceuticals and organic chemists. They considered as valuable synthons because of the inherent reactivity of the inbuilt pyran ring. Recently, it has been reported such compounds possess a broad spectrum of biological and pharmaceutical properties such as agricultural bactericide effects, photodynamic therapy, antiviral and anti-inflammatory activities (Hilderbrand & Weissleder 2007; Shchekotikhin, & Nikolaeva 2006; Fan *et al.* 2005).

Following to our recent study (Mohamed *et al.* 2012) on synthesis and structure characterization of tetrahydro xanthenones for biological investigation, herein we report the synthesis and crystal structure of the title compound.

In the title compound (I), (Fig. 1), the 4*H*-pyran ring (O1/C1/C6/C7/C12/C13) of the xanthene ring system (O1/C1–C13) is almost planar with a maximum deviation from the mean plane of 0.040 (3) Å for C13 and the cyclohexene ring (C7–C12) adopts a sofa conformation [puckering parameters (Cremer & Pople, 1975): Q_T_ = 0.457 (3) Å, θ = 121.7 (4) °, φ = 297.3 (5) °]. The cyclohexene ring (C14–C19) attached to the xanthene system is not planar: the puckering parameters of this ring are Q_T_ = 0.427 (3) Å, θ = 55.0 (4) ° and φ = 164.4 (6) °. The values of the bond lengths and bond angles are comparable with those of the related structures previously reported (Abdelhamid *et al.*, 2011; Mohamed *et al.*, 2011; Reddy *et al.*, 2009; Çelik *et al.*, 2009).

The crystal structure is stabilized by intermolecular O—H···O and C—H···O hydrogen bonds (Table 1 and Fig. 2).

### Experimental

The title compound (I) has been prepared from reaction of 1 mmol (201 mg) 5-bromo-2-hydroxybenzaldehyde with 1 mmol (140 mg) dimedone in presence of either (4-aminophenyl)methanol or TRIZMA (tris(hydroxymethyl)aminomethane) as a catalyst in 50 ml e thanol at 351 K. The reaction was monitored by TLC till completion after 4 h then left to cool at ambient temperature. The reaction mixture was concentrated under vacuum and the solid formed product was collected and dried using Buckner funnel then recrystallized from ethanol (82% yield; m.p. 521 K). Pure crystals suitable for X-ray structure analysis were obtained by slow evaporation method using ethanol as a solvent.

### Refinement

The hydroxyl H atom was located from a difference Fourier map and refined with a distance restraint of O–H 0.82±0.02. Å. Temperature factor was fixed at 1.5 times the isotropic value of the parent O atom. The hydrogen atoms at C were located geometrically and refined using a riding model with C—H = 0.95 Å (aromatic), 0.98 Å. (methyl), 0.99 Å (methylene) and 1.00 Å (methine), with *U*_iso_(H) = 1.2*U*_eq_ (aromatic, methine, methylene) and *U*_iso_(H) = 1.5*U*_eq_ (methyl).

### Crystal data

**Table:**

C_23_H_25_BrO_4_	*F*(000) = 1840
*M**_r_* = 445.33	*D*_x_ = 1.486 Mg m^−^^3^
Orthorhombic, *P**b**c**a*	Cu *K*α radiation, λ = 1.54178 Å
Hall symbol: -P 2ac 2ab	Cell parameters from 18929 reflections
*a* = 15.6869 (4) Å	θ = 1–66°
*b* = 11.0215 (2) Å	µ = 3.04 mm^−^^1^
*c* = 23.0217 (16) Å	*T* = 150 K
*V* = 3980.3 (3) Å^3^	Needle, yellow
*Z* = 8	0.12 × 0.08 × 0.02 mm

### Data collection

**Table:**

Rigaku RAPID II diffractometer	2728 reflections with *I* > 2σ(*I*)
Confocal optics monochromator	*R*_int_ = 0.044
ω scans	θ_max_ = 66.6°, θ_min_ = 3.8°
Absorption correction: multi-scan (*CrystalClear*; Rigaku, 2001)	*h* = −9→18
*T*_min_ = 0.712, *T*_max_ = 0.942	*k* = −13→10
17325 measured reflections	*l* = −27→24
3507 independent reflections	

### Refinement

**Table:**

Refinement on *F*^2^	Primary atom site location: structure-invariant direct methods
Least-squares matrix: full	Secondary atom site location: difference Fourier map
*R*[*F*^2^ > 2σ(*F*^2^)] = 0.042	Hydrogen site location: inferred from neighbouring sites
*wR*(*F*^2^) = 0.107	H atoms treated by a mixture of independent and constrained refinement
*S* = 1.07	*w* = 1/[σ^2^(*F*_o_^2^) + (0.0414*P*)^2^ + 5.2275*P*] where *P* = (*F*_o_^2^ + 2*F*_c_^2^)/3
3507 reflections	(Δ/σ)_max_ < 0.001
260 parameters	Δρ_max_ = 0.46 e Å^−^^3^
1 restraint	Δρ_min_ = −0.49 e Å^−^^3^

### Special details

**Table:**

Geometry. Bond distances, angles *etc*. have been calculated using the rounded fractional coordinates. All su's are estimated from the variances of the (full) variance-covariance matrix. The cell e.s.d.'s are taken into account in the estimation of distances, angles and torsion angles
Refinement. Refinement on *F*^2^ for ALL reflections except those flagged by the user for potential systematic errors. Weighted *R*-factors *wR* and all goodnesses of fit *S* are based on *F*^2^, conventional *R*-factors *R* are based on *F*, with *F* set to zero for negative *F*^2^. The observed criterion of *F*^2^ > σ(*F*^2^) is used only for calculating -*R*-factor-obs *etc*. and is not relevant to the choice of reflections for refinement. *R*-factors based on *F*^2^ are statistically about twice as large as those based on *F*, and *R*-factors based on ALL data will be even larger.

### Fractional atomic coordinates and isotropic or equivalent isotropic displacement parameters (Å^2^)

**Table:**

	*x*	*y*	*z*	*U*_iso_*/*U*_eq_	
Br1	0.07678 (2)	0.50005 (3)	0.19670 (1)	0.0308 (1)	
O1	0.01721 (13)	0.28166 (19)	−0.03953 (8)	0.0236 (6)	
O2	−0.16483 (15)	−0.0333 (2)	0.02080 (10)	0.0333 (8)	
O3	−0.08661 (14)	0.0562 (2)	0.16973 (10)	0.0344 (8)	
O4	−0.20527 (13)	0.3144 (2)	0.03091 (9)	0.0277 (7)	
C1	−0.00638 (18)	0.2718 (3)	0.06462 (13)	0.0199 (9)	
C2	0.01035 (19)	0.3253 (3)	0.11834 (13)	0.0242 (10)	
C3	0.06030 (19)	0.4281 (3)	0.12230 (13)	0.0257 (10)	
C4	0.0976 (2)	0.4792 (3)	0.07358 (14)	0.0269 (10)	
C5	0.08353 (19)	0.4256 (3)	0.02032 (14)	0.0251 (10)	
C6	0.03110 (18)	0.3246 (3)	0.01653 (12)	0.0206 (9)	
C7	−0.04024 (18)	0.1897 (3)	−0.04743 (13)	0.0206 (9)	
C8	−0.05102 (19)	0.1632 (3)	−0.11080 (12)	0.0229 (9)	
C9	−0.13785 (19)	0.1051 (3)	−0.12419 (13)	0.0261 (10)	
C10	−0.1512 (2)	0.0005 (3)	−0.08121 (13)	0.0250 (10)	
C11	−0.13510 (19)	0.0305 (3)	−0.01845 (13)	0.0242 (9)	
C12	−0.07972 (18)	0.1326 (3)	−0.00369 (13)	0.0196 (9)	
C13	−0.06495 (18)	0.1619 (3)	0.05977 (12)	0.0198 (9)	
C14	−0.14483 (18)	0.1801 (3)	0.09634 (12)	0.0215 (9)	
C15	−0.14520 (19)	0.1221 (3)	0.15319 (13)	0.0249 (9)	
C16	−0.2167 (2)	0.1536 (3)	0.19457 (13)	0.0294 (10)	
C17	−0.30235 (19)	0.1796 (3)	0.16603 (13)	0.0228 (9)	
C18	−0.28827 (19)	0.2733 (3)	0.11776 (13)	0.0229 (9)	
C19	−0.20994 (19)	0.2527 (3)	0.08139 (13)	0.0211 (9)	
C91	−0.1364 (2)	0.0560 (3)	−0.18629 (13)	0.0339 (11)	
C92	−0.2094 (2)	0.1994 (3)	−0.11809 (15)	0.0306 (11)	
C171	−0.3643 (2)	0.2305 (3)	0.21100 (15)	0.0341 (11)	
C172	−0.3390 (2)	0.0633 (3)	0.14004 (15)	0.0315 (11)	
H2	−0.01290	0.29060	0.15260	0.0290*	
H4	0.13220	0.54960	0.07680	0.0320*	
H4O	−0.2481 (16)	0.353 (3)	0.0282 (15)	0.0370*	
H5	0.10960	0.45770	−0.01360	0.0300*	
H8A	−0.00510	0.10770	−0.12360	0.0280*	
H8B	−0.04550	0.23960	−0.13310	0.0280*	
H10A	−0.21060	−0.02860	−0.08500	0.0300*	
H10B	−0.11310	−0.06720	−0.09240	0.0300*	
H13	−0.03350	0.09150	0.07690	0.0240*	
H16A	−0.19950	0.22580	0.21730	0.0350*	
H16B	−0.22400	0.08560	0.22220	0.0350*	
H17A	−0.37500	0.16920	0.24100	0.0510*	
H17B	−0.41820	0.25180	0.19190	0.0510*	
H17C	−0.33960	0.30300	0.22890	0.0510*	
H17D	−0.34360	0.00140	0.17040	0.0470*	
H17E	−0.30120	0.03400	0.10910	0.0470*	
H17F	−0.39560	0.07990	0.12390	0.0470*	
H18A	−0.33870	0.27310	0.09190	0.0280*	
H18B	−0.28440	0.35480	0.13570	0.0280*	
H91A	−0.12660	0.12300	−0.21350	0.0510*	
H91B	−0.19110	0.01720	−0.19500	0.0510*	
H91C	−0.09040	−0.00370	−0.19010	0.0510*	
H92A	−0.19830	0.26770	−0.14430	0.0460*	
H92B	−0.21150	0.22870	−0.07790	0.0460*	
H92C	−0.26410	0.16200	−0.12820	0.0460*	

### Atomic displacement parameters (Å^2^)

**Table:**

	*U*^11^	*U*^22^	*U*^33^	*U*^12^	*U*^13^	*U*^23^
Br1	0.0299 (2)	0.0320 (2)	0.0305 (2)	−0.0014 (2)	−0.0083 (1)	−0.0056 (1)
O1	0.0219 (11)	0.0251 (12)	0.0237 (10)	−0.0080 (10)	0.0012 (9)	−0.0007 (9)
O2	0.0342 (13)	0.0301 (13)	0.0356 (13)	−0.0122 (11)	0.0017 (11)	0.0040 (10)
O3	0.0290 (12)	0.0449 (16)	0.0292 (12)	0.0110 (12)	0.0030 (10)	0.0121 (11)
O4	0.0230 (11)	0.0304 (14)	0.0298 (11)	0.0080 (10)	0.0034 (10)	0.0097 (10)
C1	0.0140 (14)	0.0170 (16)	0.0286 (15)	0.0020 (12)	−0.0039 (12)	0.0004 (12)
C2	0.0201 (15)	0.0278 (19)	0.0246 (16)	0.0025 (14)	−0.0041 (13)	0.0000 (13)
C3	0.0203 (15)	0.0274 (19)	0.0294 (16)	0.0031 (14)	−0.0072 (13)	−0.0052 (14)
C4	0.0175 (15)	0.027 (2)	0.0361 (18)	−0.0034 (14)	−0.0038 (13)	0.0018 (14)
C5	0.0194 (15)	0.0262 (19)	0.0298 (16)	−0.0042 (14)	−0.0012 (13)	0.0005 (14)
C6	0.0146 (14)	0.0227 (17)	0.0244 (15)	−0.0016 (13)	−0.0005 (12)	−0.0015 (13)
C7	0.0156 (14)	0.0189 (17)	0.0274 (15)	0.0018 (13)	−0.0022 (12)	−0.0021 (13)
C8	0.0197 (15)	0.0247 (18)	0.0244 (15)	−0.0020 (14)	0.0010 (12)	−0.0014 (13)
C9	0.0212 (15)	0.0275 (19)	0.0295 (16)	−0.0036 (14)	−0.0035 (13)	−0.0022 (14)
C10	0.0208 (16)	0.0229 (18)	0.0312 (17)	−0.0041 (14)	−0.0024 (13)	−0.0030 (13)
C11	0.0196 (15)	0.0231 (18)	0.0299 (16)	−0.0008 (14)	−0.0018 (13)	0.0015 (13)
C12	0.0153 (14)	0.0169 (16)	0.0266 (15)	−0.0001 (12)	0.0009 (12)	0.0002 (12)
C13	0.0157 (14)	0.0202 (17)	0.0235 (15)	0.0012 (13)	−0.0020 (12)	0.0029 (12)
C14	0.0170 (15)	0.0218 (17)	0.0256 (15)	−0.0004 (13)	−0.0034 (12)	0.0032 (13)
C15	0.0212 (15)	0.0271 (18)	0.0264 (16)	0.0006 (15)	−0.0017 (13)	0.0043 (13)
C16	0.0242 (17)	0.039 (2)	0.0250 (16)	0.0009 (16)	0.0011 (13)	0.0027 (14)
C17	0.0196 (15)	0.0239 (18)	0.0248 (15)	0.0030 (13)	0.0020 (13)	0.0031 (13)
C18	0.0201 (15)	0.0237 (18)	0.0250 (16)	0.0023 (14)	0.0002 (13)	0.0018 (13)
C19	0.0219 (15)	0.0185 (16)	0.0229 (15)	−0.0033 (13)	0.0002 (13)	0.0032 (12)
C91	0.038 (2)	0.034 (2)	0.0298 (17)	−0.0031 (18)	−0.0031 (15)	−0.0053 (15)
C92	0.0225 (16)	0.031 (2)	0.0384 (19)	0.0004 (15)	−0.0053 (14)	0.0039 (15)
C171	0.0276 (18)	0.040 (2)	0.0347 (18)	0.0081 (17)	0.0074 (15)	0.0039 (16)
C172	0.0234 (17)	0.031 (2)	0.0400 (19)	−0.0016 (16)	−0.0006 (14)	0.0069 (16)

### Geometric parameters (Å, º)

**Table:**

Br1—C3	1.905 (3)	C17—C18	1.533 (4)
O1—C6	1.392 (3)	C17—C172	1.527 (5)
O1—C7	1.368 (4)	C17—C171	1.527 (4)
O2—C11	1.236 (4)	C18—C19	1.504 (4)
O3—C15	1.232 (4)	C2—H2	0.9500
O4—C19	1.349 (4)	C4—H4	0.9500
O4—H4O	0.80 (3)	C5—H5	0.9500
C1—C6	1.382 (4)	C8—H8A	0.9900
C1—C13	1.524 (4)	C8—H8B	0.9900
C1—C2	1.395 (4)	C10—H10A	0.9900
C2—C3	1.381 (5)	C10—H10B	0.9900
C3—C4	1.385 (4)	C13—H13	1.0000
C4—C5	1.379 (5)	C16—H16A	0.9900
C5—C6	1.387 (4)	C16—H16B	0.9900
C7—C12	1.339 (4)	C18—H18A	0.9900
C7—C8	1.497 (4)	C18—H18B	0.9900
C8—C9	1.536 (4)	C91—H91A	0.9800
C9—C91	1.529 (4)	C91—H91B	0.9800
C9—C92	1.536 (4)	C91—H91C	0.9800
C9—C10	1.534 (4)	C92—H92A	0.9800
C10—C11	1.504 (4)	C92—H92B	0.9800
C11—C12	1.462 (4)	C92—H92C	0.9800
C12—C13	1.514 (4)	C171—H17A	0.9800
C13—C14	1.523 (4)	C171—H17B	0.9800
C14—C15	1.457 (4)	C171—H17C	0.9800
C14—C19	1.342 (4)	C172—H17D	0.9800
C15—C16	1.512 (4)	C172—H17E	0.9800
C16—C17	1.523 (4)	C172—H17F	0.9800
			
C6—O1—C7	118.6 (2)	C3—C2—H2	120.00
C19—O4—H4O	107 (2)	C3—C4—H4	121.00
C2—C1—C13	120.9 (3)	C5—C4—H4	121.00
C6—C1—C13	122.2 (3)	C4—C5—H5	120.00
C2—C1—C6	116.9 (3)	C6—C5—H5	120.00
C1—C2—C3	120.8 (3)	C7—C8—H8A	109.00
Br1—C3—C4	120.1 (2)	C7—C8—H8B	109.00
C2—C3—C4	121.3 (3)	C9—C8—H8A	109.00
Br1—C3—C2	118.6 (2)	C9—C8—H8B	109.00
C3—C4—C5	118.6 (3)	H8A—C8—H8B	108.00
C4—C5—C6	119.7 (3)	C9—C10—H10A	108.00
O1—C6—C1	122.2 (3)	C9—C10—H10B	108.00
O1—C6—C5	115.1 (3)	C11—C10—H10A	108.00
C1—C6—C5	122.7 (3)	C11—C10—H10B	108.00
O1—C7—C12	123.6 (3)	H10A—C10—H10B	108.00
C8—C7—C12	126.1 (3)	C1—C13—H13	107.00
O1—C7—C8	110.4 (2)	C12—C13—H13	107.00
C7—C8—C9	112.1 (2)	C14—C13—H13	107.00
C8—C9—C10	107.8 (2)	C15—C16—H16A	108.00
C8—C9—C91	108.8 (2)	C15—C16—H16B	108.00
C10—C9—C91	109.8 (3)	C17—C16—H16A	108.00
C10—C9—C92	110.5 (3)	C17—C16—H16B	109.00
C91—C9—C92	109.6 (3)	H16A—C16—H16B	107.00
C8—C9—C92	110.3 (3)	C17—C18—H18A	109.00
C9—C10—C11	115.6 (3)	C17—C18—H18B	109.00
O2—C11—C10	120.9 (3)	C19—C18—H18A	109.00
C10—C11—C12	119.5 (3)	C19—C18—H18B	109.00
O2—C11—C12	119.5 (3)	H18A—C18—H18B	108.00
C7—C12—C11	117.5 (3)	C9—C91—H91A	109.00
C11—C12—C13	118.7 (3)	C9—C91—H91B	109.00
C7—C12—C13	123.7 (3)	C9—C91—H91C	109.00
C1—C13—C12	109.4 (2)	H91A—C91—H91B	110.00
C1—C13—C14	110.5 (3)	H91A—C91—H91C	109.00
C12—C13—C14	115.8 (2)	H91B—C91—H91C	109.00
C13—C14—C15	116.3 (3)	C9—C92—H92A	109.00
C13—C14—C19	124.3 (3)	C9—C92—H92B	109.00
C15—C14—C19	119.3 (3)	C9—C92—H92C	109.00
O3—C15—C16	119.6 (3)	H92A—C92—H92B	110.00
C14—C15—C16	117.9 (3)	H92A—C92—H92C	109.00
O3—C15—C14	122.3 (3)	H92B—C92—H92C	109.00
C15—C16—C17	115.2 (2)	C17—C171—H17A	109.00
C16—C17—C171	109.8 (2)	C17—C171—H17B	109.00
C16—C17—C172	110.1 (3)	C17—C171—H17C	109.00
C16—C17—C18	108.2 (2)	H17A—C171—H17B	109.00
C18—C17—C172	109.6 (3)	H17A—C171—H17C	109.00
C171—C17—C172	109.5 (3)	H17B—C171—H17C	110.00
C18—C17—C171	109.6 (3)	C17—C172—H17D	109.00
C17—C18—C19	114.8 (3)	C17—C172—H17E	109.00
O4—C19—C18	116.6 (3)	C17—C172—H17F	109.00
C14—C19—C18	124.7 (3)	H17D—C172—H17E	109.00
O4—C19—C14	118.7 (3)	H17D—C172—H17F	110.00
C1—C2—H2	120.00	H17E—C172—H17F	109.00
			
C7—O1—C6—C5	−173.8 (3)	C9—C10—C11—O2	160.4 (3)
C6—O1—C7—C8	175.6 (2)	C9—C10—C11—C12	−23.0 (4)
C7—O1—C6—C1	4.8 (4)	O2—C11—C12—C7	171.7 (3)
C6—O1—C7—C12	−4.8 (4)	O2—C11—C12—C13	−3.8 (4)
C6—C1—C2—C3	1.7 (4)	C10—C11—C12—C7	−5.0 (4)
C13—C1—C2—C3	−177.0 (3)	C10—C11—C12—C13	179.5 (3)
C2—C1—C13—C12	173.4 (3)	C7—C12—C13—C1	5.4 (4)
C2—C1—C13—C14	44.7 (4)	C7—C12—C13—C14	131.1 (3)
C6—C1—C13—C12	−5.3 (4)	C11—C12—C13—C1	−179.4 (3)
C6—C1—C13—C14	−134.0 (3)	C11—C12—C13—C14	−53.7 (4)
C2—C1—C6—O1	−178.1 (3)	C1—C13—C14—C15	−98.9 (3)
C2—C1—C6—C5	0.4 (5)	C1—C13—C14—C19	75.5 (4)
C13—C1—C6—O1	0.6 (5)	C12—C13—C14—C15	135.9 (3)
C13—C1—C6—C5	179.1 (3)	C12—C13—C14—C19	−49.6 (4)
C1—C2—C3—Br1	177.5 (2)	C13—C14—C15—O3	−4.1 (5)
C1—C2—C3—C4	−2.1 (5)	C13—C14—C15—C16	170.6 (3)
Br1—C3—C4—C5	−179.3 (2)	C19—C14—C15—O3	−178.8 (3)
C2—C3—C4—C5	0.4 (5)	C19—C14—C15—C16	−4.1 (4)
C3—C4—C5—C6	1.7 (5)	C13—C14—C19—O4	−0.2 (5)
C4—C5—C6—O1	176.5 (3)	C13—C14—C19—C18	−178.7 (3)
C4—C5—C6—C1	−2.1 (5)	C15—C14—C19—O4	174.1 (3)
O1—C7—C8—C9	−155.2 (3)	C15—C14—C19—C18	−4.4 (5)
C12—C7—C8—C9	25.3 (4)	O3—C15—C16—C17	−151.6 (3)
O1—C7—C12—C11	−175.9 (3)	C14—C15—C16—C17	33.6 (4)
O1—C7—C12—C13	−0.7 (5)	C15—C16—C17—C18	−50.9 (4)
C8—C7—C12—C11	3.5 (5)	C15—C16—C17—C171	−170.5 (3)
C8—C7—C12—C13	178.8 (3)	C15—C16—C17—C172	68.9 (3)
C7—C8—C9—C10	−48.8 (3)	C16—C17—C18—C19	42.1 (3)
C7—C8—C9—C91	−167.8 (3)	C171—C17—C18—C19	161.8 (3)
C7—C8—C9—C92	71.9 (3)	C172—C17—C18—C19	−78.0 (3)
C8—C9—C10—C11	48.9 (3)	C17—C18—C19—O4	165.1 (3)
C91—C9—C10—C11	167.2 (3)	C17—C18—C19—C14	−16.3 (4)
C92—C9—C10—C11	−71.7 (3)		

### Hydrogen-bond geometry (Å, º)

**Table:**

*D*—H···*A*	*D*—H	H···*A*	*D*···*A*	*D*—H···*A*
O4—H4*O*···O2^i^	0.80 (3)	1.86 (3)	2.650 (3)	170 (3)
C8—H8*A*···O3^ii^	0.99	2.54	3.514 (4)	167
C13—H13···O3	1.00	2.33	2.807 (4)	108

Symmetry codes: (i) −*x*−1/2, *y*+1/2, *z*; (ii) −*x*, −*y*, −*z*.
